# Adsorption and Sensing Performances of Pristine and Au-Decorated Gallium Nitride Monolayer to Noxious Gas Molecules: A DFT Investigation

**DOI:** 10.3389/fchem.2022.898154

**Published:** 2022-05-12

**Authors:** Zhihui Li, Lufen Jia, Jianxing Chen, Xiaosen Cui, Qu Zhou

**Affiliations:** College of Engineering and Technology, Southwest University, Chongqing, China

**Keywords:** NO, Cl_2_, O_3_, DFT, Au-decorated GaN, gas adsorption

## Abstract

In this study, the adsorption of noxious gas molecules (NO, Cl_2_, and O_3_) on GaN and Au-decorated GaN was systematically scrutinized, and the adsorption energy, bond length, charge, density of state (DOS), partial density of state (PDOS), electron deformation density (EDD), and orbitals were analyzed by the density functional theory (DFT) method. It is found that the interaction between NO and pristine GaN is physical adsorption, while GaN chemically reacts with Cl_2_ and O_3_. These observations suggest that pristine GaN may be a candidate for the detection of Cl_2_ and O_3_. The highly activated Au-decorated GaN can enhance the adsorption performance toward NO and convert the physical adsorption for NO into chemical adsorption, explaining the fact that precious metal doping is essential for regulating the electronic properties of the substrate material. This further confirms the well-established role of Au-decorated GaN in NO gas-sensing applications. In addition, the adsorption performance of Au-decorated GaN for Cl_2_ and O_3_ molecules is highly improved, which provides guidance to scavenge toxic gases such as Cl_2_ and O_3_ by the Au-decorated GaN material.

## Introduction

To quest for the excellent and unique electronic properties of materials, semiconductor materials have gradually become one of the global research hotspots in the realm of sensor components and optoelectronic devices (Zhou et al., 2018a; Wei et al., 2020). Gallium nitride (GaN), the third generation semiconductor material, possesses exciting traits including high thermal conductivity, remarkable chemical stability, and wide direct band gap (Sun et al., 2017; Cui et al., 2020). More importantly, with the deepening of research on two-dimensional (2D) materials, a volume of 2D materials for gas adsorption and sensing has been explored in many realms. At the same time, 2D GaN, because of its unique graphite-like honeycomb structure, large surface area, and high electron mobility, has also gradually motivated the hunt for the GaN monolayer in gas adsorption and sensing fields. (Onen et al., 2017; Liu et al., 2021; Ren et al., 2021; Wang et al., 2021). For instance, Chen et al. (2019) calculated and studied the 2D GaN monolayer decorated with Fe and Mn metals by first principles and found that it is a promising new gas-warning material for toxic gas molecules. Cui et al. (2020) explored the adsorption of noxious gas molecules (CO, NH_3_, NO, and NO_2_) by intrinsic and defective GaN, indicating that the structure has a certain potential in sensing ability. For the foreseeable future, the 2D GaN monolayer holds great potential in the application of nanoscale gas sensor devices for noxious gas.

In recent years, large fossil fuel consumption and motor vehicle exhaust emissions have led to a surge in emissions of pollutants such as NO and O_3_ with the rapid development of urbanization and industrialization, thus aggravating the air pollution situation. When the concentration of ozone in the air is high, it is easy to cause respiratory discomfort, bronchitis, chronic lung disease, and even death in serious cases (Pryor et al., 1995). In addition, NO is also one of the main reasons causing adverse effects such as acid rain, photochemical smoke, and the ozone layer depletion, which is harmful to the environment and human health (Roy and Baiker, 2009; Saeidi et al., 2021). As a toxic gas produced in industrial production, Cl_2_ can damage the organs and systems of the whole body and have many harmful effects on the human body (Bender et al., 2001; Azimi and Tazikeh-Lemeski, 2018). Therefore, one of the current urgent problems to be solved is to monitor the concentration of the toxic gases (Lee and Lee, 2001). Currently, there are a large number of studies on monitoring NO (Gao, 2017), O_3_ (Kamalinahad et al., 2016; Rad et al., 2016; Chaabene et al., 2021), and Cl_2_ (Li et al., 2006; Beheshtian et al., 2012; Wen et al., 2020). For example, Saeidi et al. (2021) have pointed out that Si–N_4_-embedded graphene can remove harmful NO from the atmospheric environment by electrochemical reduction methods. Furthermore, Mi et al. (2021) reported that Ag_3_–WSe_2_ may perform as a promising gas sensor material to monitor Cl_2_, NH_3_, and NO_2_ toxic gases, explaining the cluster metal doping superiority. Furthermore, as reported, noble metals such as Au, Ag, Pt, and Pd doping can improve the properties of the adsorption response to the target gas molecules by enhancing the electron transfer between the interfaces (Li et al., 2020). Noble metals, as an activator, change the electronic state of the pristine material and can greatly enhance the selectivity and response speed of gas-sensitive materials and reduce their working temperature (Zhou et al., 2018b). For example, Peng et al. (2021), by comparing the adsorption with the intrinsic InN, found that the absorption characteristic for Au–InN to the SF_6_-decomposed gases remains superior to InN, reflecting the good electron mobility of Au doping. Moreover, Dong et al. (2011) reported the response of noble metal Pt-decorated SnO_2_ nanofibers to gas H_2_S and obtained that the gas-sensitive response rate of 0.08wt% Pt-decorated SnO_2_ nanofibers to H_2_S was 25.9–40.6 times higher than that of pristine SnO_2_ nanofibers, which proved that noble metal doping can greatly improve the gas-sensitive response. However, there are few theoretical studies on 2D GaN material adsorption to NO, O_3_, and Cl_2_ gases, and GaN decorated with Au is unexplored for the three noxious gas molecules.

In the paper, a DFT method was employed to investigate the adsorption performances and sensing capabilities of the 2D GaN substrate to toxic gas molecules (NO, O_3_, and Cl_2_). Also, Au doping was explored as a mean to improve the adsorption properties of GaN substrate for NO, O_3_, and Cl_2_ gases.

## Computational Details

All calculations in this study were performed using Dmol3, a quantum chemistry module software package of Materials Studio (Qian et al., 2020). The simulation calculations constructed in this study are based on DFT (Delley, 1990; Ni et al., 2020). The DFT method is a mutipurpose technique to investigate the interface of nanoparticles and electronic structure (Mousavi-Khoshdel et al., 2016; Jahanbakhsh-Bonab et al., 2021). The lattice parameters of the 4 × 4 × 1 perfect GaN crystal plane supercell model are a = b = 12.76Å and C = 20.65Å, and a vacuum layer with a thickness of 20Å was added to avoid the influence of adjacent periodic images (Gao et al., 2020). Generalized gradient approximation (GGA) is a functional based on the non-uniform electronic gas model (Grimme, 2006; Wang et al., 2019). This approximation deals with the exchange correlation energy, resulting in a smaller error. In addition, the Perdew–Burke–Ernzerhof (PBE) functional was mostly used for 2D material correlation calculations. Considering that PBE amplification underestimates the non-local interaction, the van der Waals-modified parameter DFT-D2 was adopted (Perdew et al., 1993; Perdew et al., 1997). Electron pseudopotential was calculated by double numerical with polarization (DNP) (Cui et al., 2019). The tolerance of convergence force, convergence displacement, convergence total energy, the self-consistent field (SCF) tolerance, and smearing of orbital occupancy were set at 2 × 10^−3^ Ha/Å, 5 × 10^−3^ Å, 1 × 10^−5^ Ha, 1 × 10^−6^ Ha, and 0.005 Ha, respectively (Ju et al., 2017). The k value of Monkhorst–Pack was set at 6 × 6 × 1, so as to accurately obtain the physicochemical parameters of the GaN system.

The position with the lowest binding energy is regarded as the optimal adsorptive position, and the binding energy is described as formula (1):

$$E_{\text{b}}=E_{\text{Au}-\text{GaN}}-E_{\text{GaN}}-E_{\text{Au}}.$$ (1)

In the formula, *E*_Au-GaN_ stands for the total energy of the optimized Au-decorated GaN structure, *E*_GaN_ is the energy of the optimized GaN, and *E*_Au_ describes the energy of the optimized Au atom.

The size of adsorption energy is usually used to judge the stability of different adsorption systems. The smaller the adsorption energy value is, the more stable the adsorption structure is and the more obvious the interface interaction is. The general form of adsorption energy can be expressed by formula (2) as follows:

$$E_{\text{ads}}=E_{\text{total}}-E_{\text{Au}-\text{GaN}}-E_{\text{gas}}.$$ (2)

Here, *E*_total_, *E*_Au-GaN_, and *E*_gas_ stand for the total energies of gas molecules on Au-decorated GaN, Au-decorated GaN, and the gas molecules NO, Cl_2_, and O_3_, respectively.

In order to know whether the gases adsorbed on the crystal surface are strong or not and to judge the stability of adsorption, the Hirshfeld charge distribution of the stable adsorption structure between gas molecules and crystal plane was calculated, and the charge transfer quantity *Q*_t_ is defined as the charge change between the crystal plane and gas molecule. The adsorption distance (*D*) is defined as the distance between the crystal surface and the atom of a gas molecule. The energy gap width of the total adsorption system is expressed by *E*_g_.

## Results and Discussion

### Establishment of NO, Cl_2_, and O_3_

**FIGURE 1:**
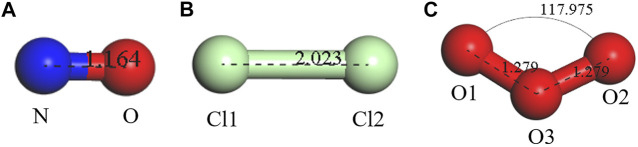
Structures of **(A)** NO, **(B)** Cl_2_, and **(C)** O_3_.

After reaching the convergence standard, the final stable and reasonable geometric structures of gas molecules (NO, Cl_2_, and O_3_), as shown in Figures 1A–C, are almost consistent with the geometric configuration of other relevant studies (Deka et al., 2014; Tabari and Farmanzadeh, 2019). Blue, red, and green spheres represent nitrogen atoms, oxygen atoms, and chlorine atoms, respectively. Obviously, NO and Cl_2_ molecules are linear, with bond lengths of 1.164 Å and 2.023 Å, respectively. In addition, it can be found that the O_3_ molecule is a plane triangle, in which O_1_–O_3_ and O_2_–O_3_ bonds are 1.279 Å, and the bond angle of O_1_–O_3_–O_2_ is 117.975°.

### Establishment of the Gallium Nitride Monolayer and Au–Gallium Nitride Monolayer

**FIGURE 2:**
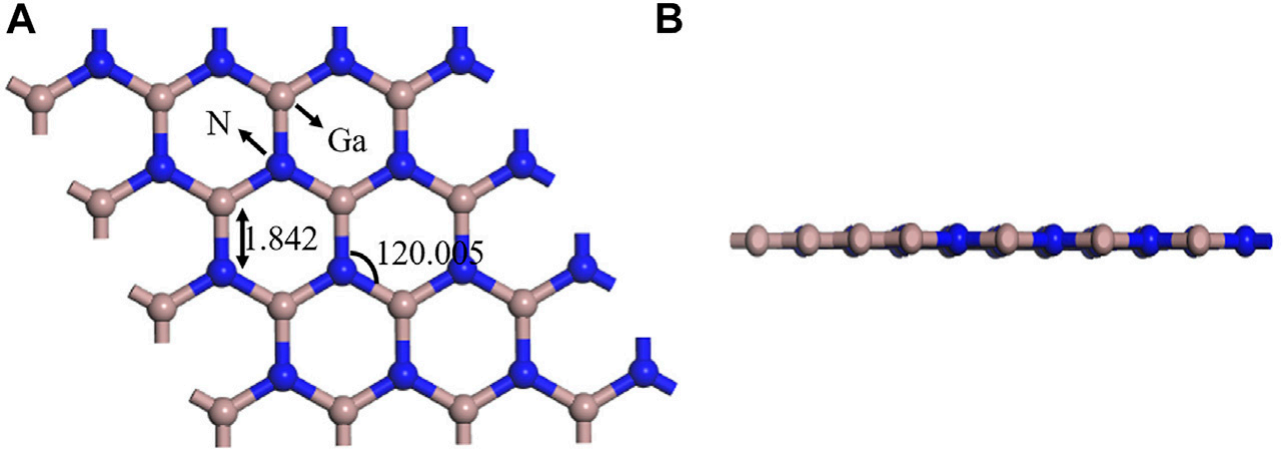
**(A)** Top view and **(B)** side view of the GaN structure.

**FIGURE 3:**
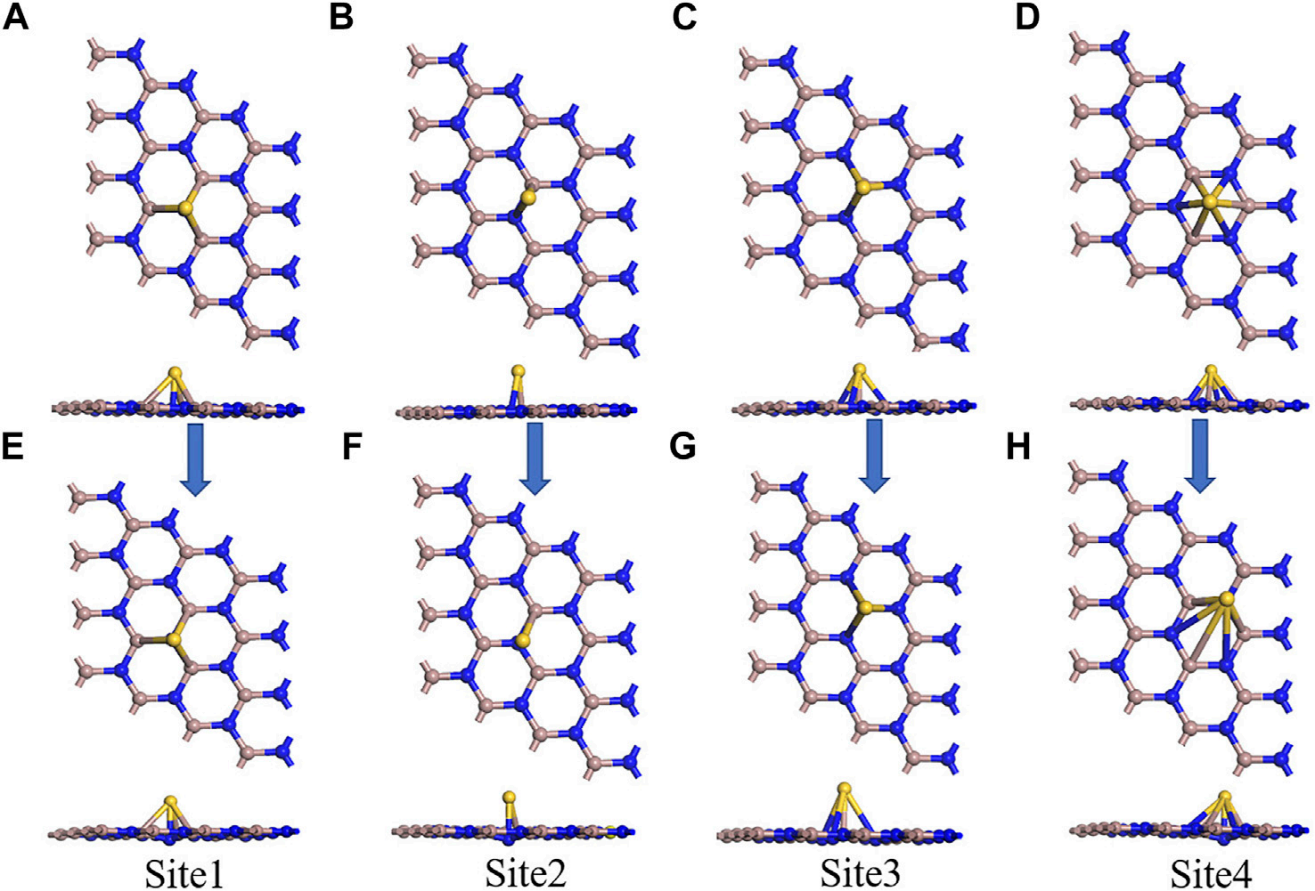
Four initial models of Au-decorated GaN. **(A)** Site 1, **(B)** site 2, **(C)** site 3, and **(D)** site 4. Four optimization models of Au-decorated GaN **(E)**Site 1, **(F)** site 2, **(G)** site 3, and **(H)** site 4.

In Figure 2, a 2D plane hexagonal honeycomb GaN structure with symmetry was obtained by optimizing the GaN monolayer. Similar to graphene, the Ga atoms of the 2D GaN connect with the surrounding N atoms to form covalent bonds. The calculated bond length and bond angle are 1.842 Å and 120.005°, respectively, which are almost in good agreement with previous reports (Jia et al., 2021). For studying adsorption properties, the GaN surface is modified using the metal Au. The Au atom is initially placed in four adsorption positions of GaN monolayer, as shown in Figure 3. Site 1 in Figure 3A and site 3 in Figure 3C indicate that the Au atom is placed vertically above the N atom and Ga atom, respectively. Site 2 in Figure 3B indicates that the Au atom is above the Ga–N bond. Site 4 in Figure 3D is said to be the hollow position of the whole hexagonal structure of GaN. It can be intuitively found that after the structure is optimized, the doping metal Au at sites 1, 2, and will eventually fall on top of the N atom nearest to the Au metal, as shown in Figures 3E,F,H. Meanwhile, we can also find a detail that the GaN surface has obvious deformation, which is reflected in the bulge of the N atom below the Au atom toward the lower part of the entire GaN plane. However, there are almost no obvious structural changes in the entire GaN plane in Figure 3G. The characteristics of structural changes directly indicate that there is no strong interaction between the base material and Au metal at the doping site 3. In addition, it can also be demonstrated from the perspective of binding energy. By comparing doping parameters in Table 1, it can be obtained that the absolute value of binding energy (-1.197 eV) at site 1 is the smallest, and the doping distance (2.535 Å) is also the smallest. Therefore, doping site 1 is the optimal doping location.

**TABLE 1:** Bond length and binding energies of the four different doping sites.

Doping site	*E*_b_ (eV)	Bond length (Å)
Site 1	−1.197	2.535 (Au–N)
Site 2	−1.197	2.540 (Au–N)
Site 3	−1.034	2.563 (Au–Ga)
Site 4	−1.143	2.538 (Au–N)

**FIGURE 4:**
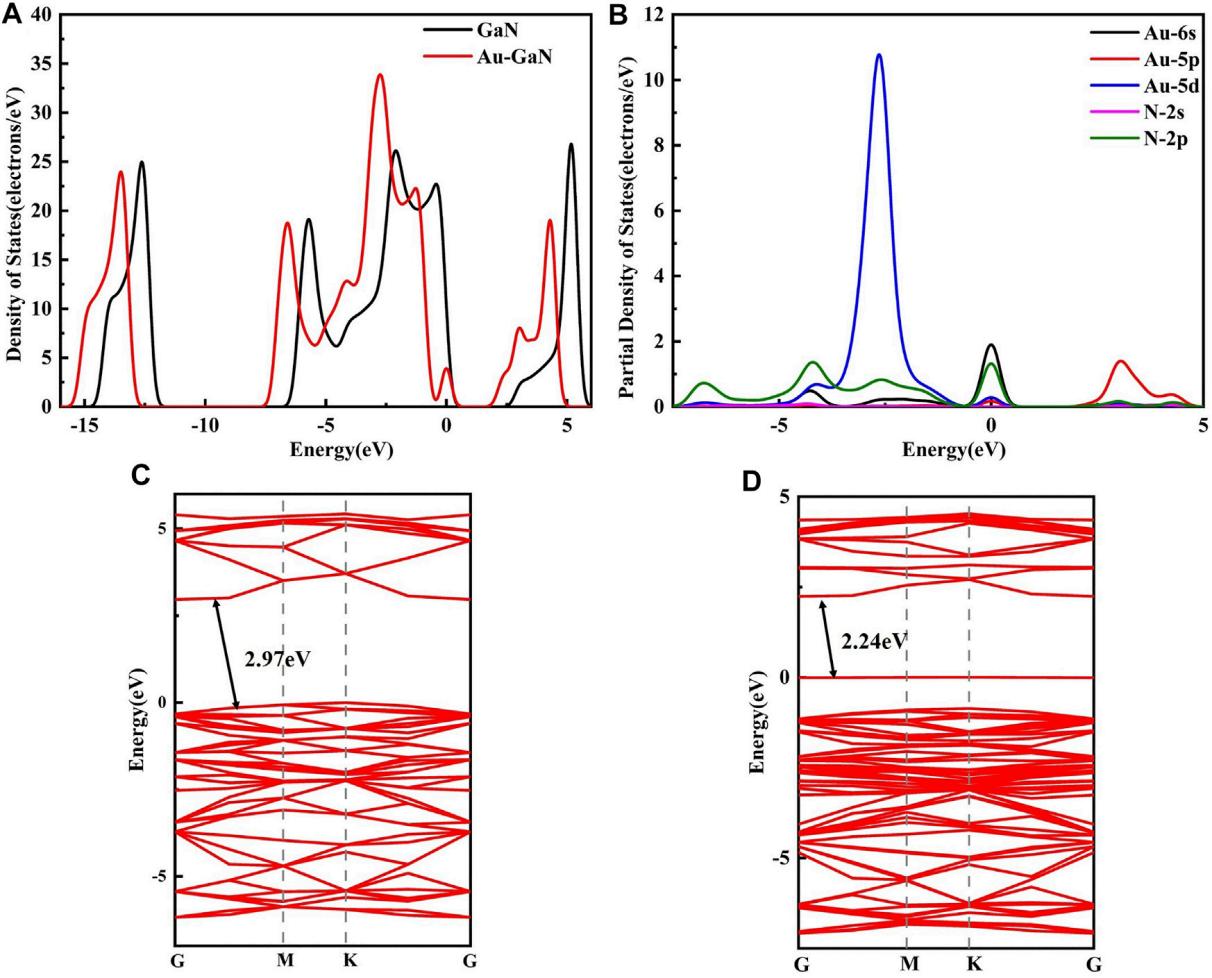
**(A)** DOS of GaN and Au–GaN monolayer, **(B)** PDOS of Au-GaN monolayer, **(C)** band structure of GaN, and **(D)** band structure of Au–GaN.

In order to understand the performance of doping or adsorption from the perspective of electronic properties, the band structures of GaN and Au–GaN, DOS, and PDOS of the related systems were introduced and calculated. From Figure 4A, the DOS of Au–GaN crystal surface is approximated to that of intrinsic GaN, indicating that the crystal structure of GaN decorated with Au does not change much. But overall DOS of the Au–GaN tends to move to the left. The peak of DOS increases at -2.58eV, and a new peak appears at the Fermi level, which is due to orbital hybridization between Au and GaN. Also, a major finding is that the doping of Au atom reduces the distance of the valence band electrons and the conduction band hole transition, reflected in the abscissa of the DOS where the intercept near the Fermi level is narrowed. As can be seen from the PDOS in Figure 4B, overlapping peaks exist for the orbital of Au and N at -5 to 0 eV. It indicates that there is strong hybridization between orbitals, so a stable Au–GaN structure is formed. Meanwhile, according to the band structures of intrinsic GaN (see in Figure 4C) and Au–GaN (see in Figure 4D), it is more intuitive that the band gap of GaN decreases obviously from 2.97eV to 2.24eV after doping Au. Therefore, Au doping reduces the band gap of the GaN crystal surface and leads to the improved conductivity of the GaN crystal surface.

### Analysis of the Adsorption Structure and Electronic Characteristics

**FIGURE 5:**
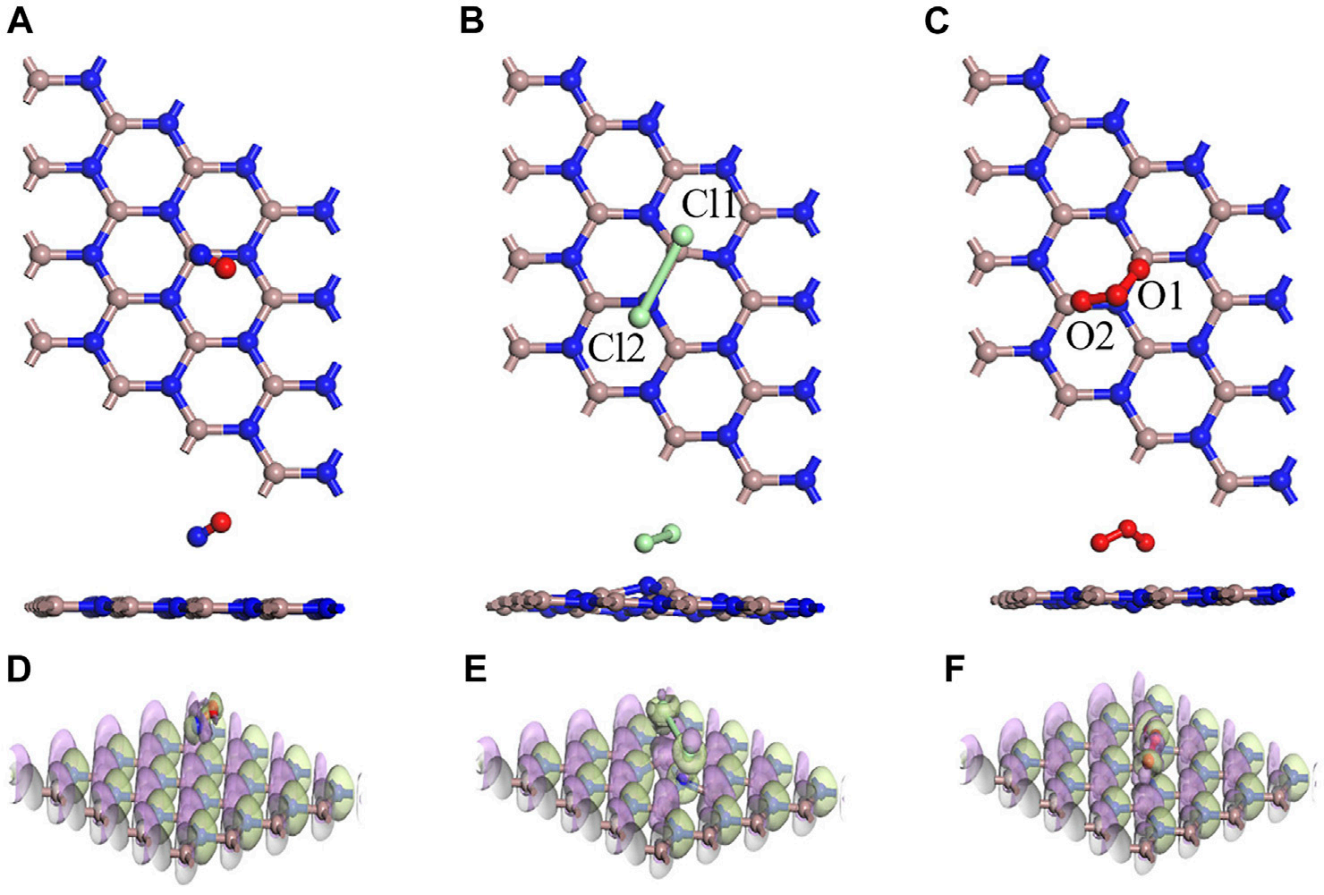
Optimized structures for **(A)** NO, **(B)** Cl_2_, and **(C)** O_3_ on the GaN surface and EDD for **(D)** NO, **(E)** Cl_2_, and **(F)** O_3_ on the GaN surface (purple regions indicate loss of electrons, and green regions represent obtained electrons in EDD).

In Figure 5, the optimal structural configurations of the three toxic gases (NO, Cl_2_, and O_3_) adsorbed on the intrinsic GaN surface are shown. During simulation calculations in this work, it is found that before the optimization of the entire adsorption system, when any atom of the NO is close to the entire GaN surface, the final stable configuration is the N atom of NO near the GaN plane. However, the whole NO adsorption system belongs to physical adsorption, which is reflected in the following aspects: the absolute value of adsorption energy is less than 0.8eV, and the length of the N–O bond is almost unchanged. In addition, in Table 2, the bond length of Cl_1_–Cl_2_ is extended from 2.023 Å to 3.321 Å, demonstrating that the Cl_2_ molecule breaks and forms Cl_1_–Ga and Cl_2_–N bonds. It can also be noted from Figure 5C that the two O atoms of the O_3_ molecules are close to the Ga atoms with an adsorption distance of 2.222 Å. By comparing the bond length and bond angle of O_3_ molecules before and after adsorption, we can also find structural changes of O_3_ molecules. Comparing with the side view of the three molecules adsorbed on the GaN surface, it can be seen intuitively that the deformation degree of the GaN surface is in the following order: Cl_2_ > O_3_ > NO. In addition, by observing Figure 7, it can also be found that the absolute value of adsorption energy for the three adsorption systems is arranged as follows: Cl_2_ > O_3_ > NO, in the same order as the amount of charge transfer. In Figures 5D–F, the EDD of the three adsorption systems is shown. The charge transfer relationship between the gas molecules and the base material can be visualized by the EDD. It can be observed that the green regions distributed around the Cl_2_ and O_3_ molecules in Figures 5E,F occupy a large space, showing the accumulation of electrons. Therefore, it is shown that the two molecules derive electrons from the GaN surface. In conclusion, Cl_2_ and O_3_ are chemisorbed to the intrinsic GaN, and the adsorption capacity is better than that of NO.

**TABLE 2:** Adsorption parameters of NO, Cl_2_, and O_3_ on the pure GaN surface.

System	*E*_ads_ (eV)	*Q*_t_ (e)	*B*_g_ (eV)	*D* (Å)	Bond length (Å)	Bond angle (°)
GaN/NO	−0.272	0.026	−0.273	2.700 (N–Ga)	1.167 (N–O)	/
GaN/Cl_2_	−1.034	−0.249	−1.986	2.224 (Cl_1_–Ga) and 1.839 (Cl_2_–N)	3.321 (Cl_1_–Cl_2_)	/
GaN/O_3_	−0.95	−0.166	−0.599	2.222 (O_1_–Ga_1_) and 2.238 (O_2_–Ga_2_)	1.339 (O_1_–O_3_) and 1.336 (O_2_–O_3_)	117.193 (O_1_–O_3_–O_2_)

**FIGURE 6:**
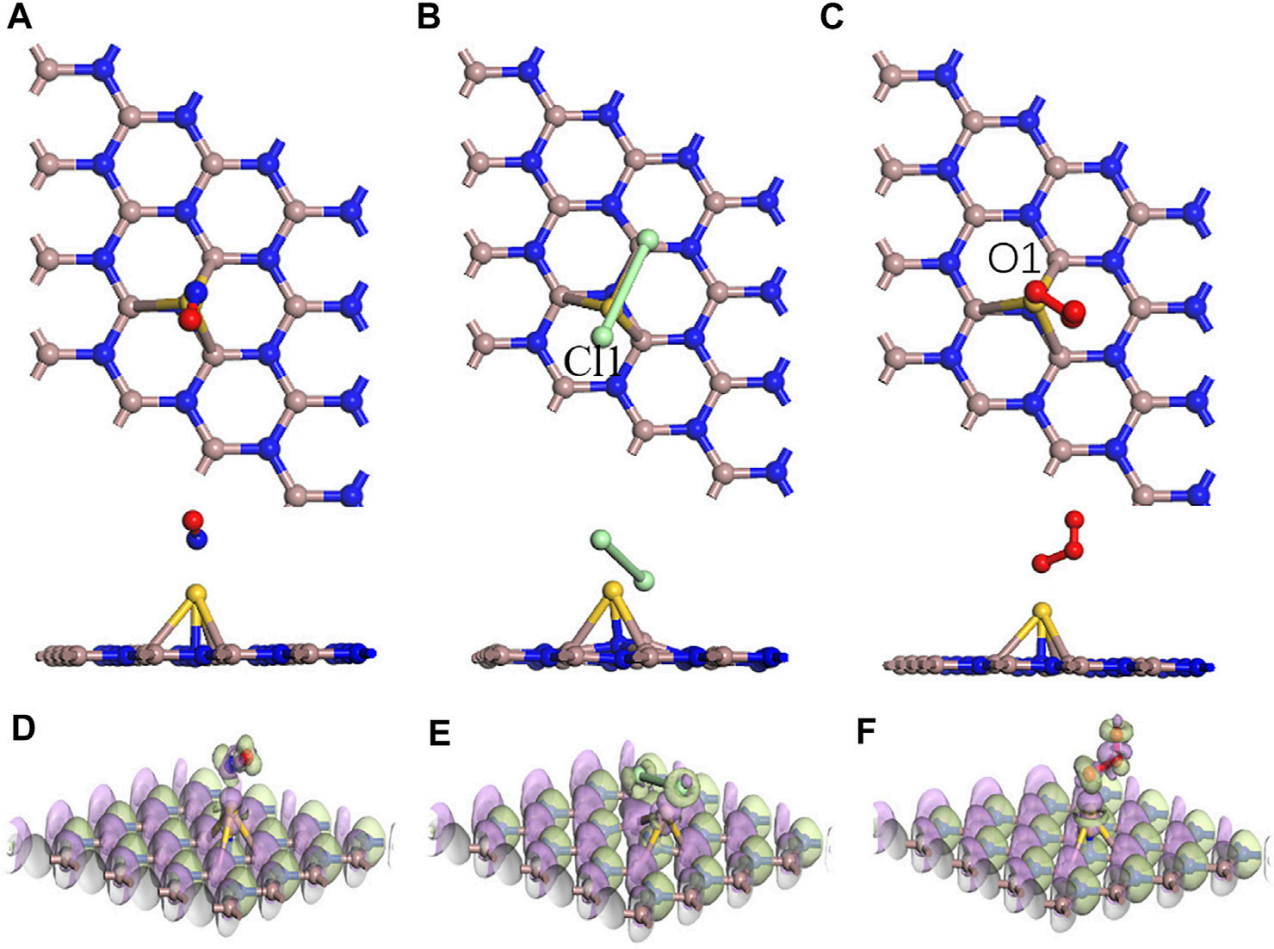
Optimized structures for **(A)** NO, **(B)** Cl_2_, and **(C)** O_3_ on the Au–GaN surface and EDD for **(D)** NO, **(E)** Cl_2_, and **(F)** O_3_ on the Au–GaN surface (purple regions indicate loss of electrons, and green regions represent obtained electrons in EDD).

**FIGURE 7:**
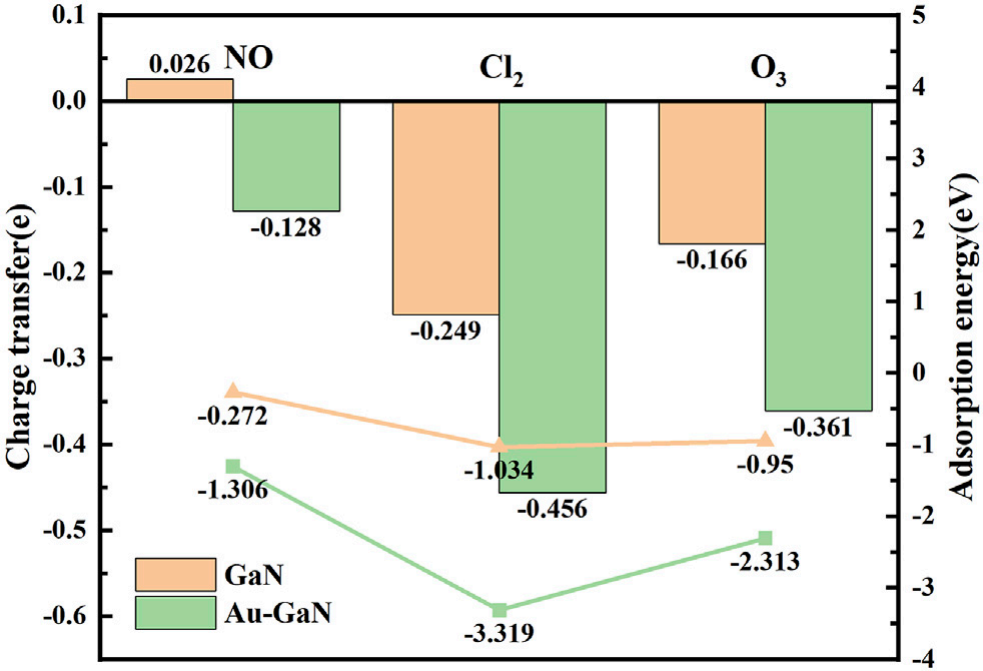
Charge transfer and adsorption energy of GaN and Au–GaN adsorption systems (the bar diagram represents the charge transfer, and the broken line diagram represents the adsorption energy).

In Figure 6, the adsorption performances were analyzed from the perspective of the geometric configuration. The Au atom is greatly shifted in Figure 6B, but the Au atoms of the other two systems are still directly above the N atoms in Figures 6A, C. The longitudinal comparison of the adsorption energy and the transfer charge can also further infer that the sensor prepared by GaN decorated with Au has the better adsorption performance for Cl_2_ in Figure 7. Compared with the three gases on intrinsic GaN, it can be found that the adsorption performance of GaN can be improved by doping with metal, which is embodied in the adsorption parameters as shown in Table 3. The adsorption type of NO adsorption on Au–GaN has been converted from physical adsorption to chemical adsorption, compared to the intrinsic adsorption of this gas. Compared with Cl_2_ adsorption in the intrinsic system, the adsorption energy increases by three times, and the Cl_1_–Cl_2_ bond length is also extended from 3.321 Å to 4.238 Å. Compared with the O_3_ adsorption in the intrinsic system from Table 2 and Table 3, the adsorption energy is doubled, the O_1_–O_3_ bond length also changes from 1.339 Å to 1.606 Å, and the angle of O_1_–O_3_–O_2_ is reduced from 117.193° to 110.7°. At the same time, it is found that the transfer charge of the three adsorption systems has also increased to different degrees in Figure 7. By comparing EDD in Figure 5D and Figure 6D, it is found that the green area around NO in Figure 6D is significantly increased, which proves that the amount of charge transfer is significantly increased, showing that electrons are obtained. In Figures 6D–F, the metal Au atom in EDD is surrounded by the purple region, indicating electron consumption in the whole adsorption process. In short, Au-GaN has a better adsorption effect than GaN.

**TABLE 3:** Adsorption parameters of NO, Cl_2_, and O_3_ on the Au–GaN surface.

System	*E*_ads_ (eV)	*Q*_t_ (e)	*B*_g_ (eV)	*D* (Å)	Bond length (Å)	Bond angle (°)
Au–GaN/NO	−1.306	−0.128	−0.762	2.099 (N-Au)	1.192 (N–O)	/
Au–GaN/Cl_2_	−3.319	−0.456	−2.776	2.291 (Cl_1_-Au)	4.238 (Cl_1_–Cl_2_)	/
Au–GaN/O_3_	−2.313	−0.361	−2.966	1.991 (O_1_-Au)	1.606 (O_1_–O_3_) and 1.254 (O_2_-O_3_)	110.700(O_1_–O_3_–O_2_)

### Analysis of DOS and Frontier Molecular Orbital Theory

**FIGURE 8:**
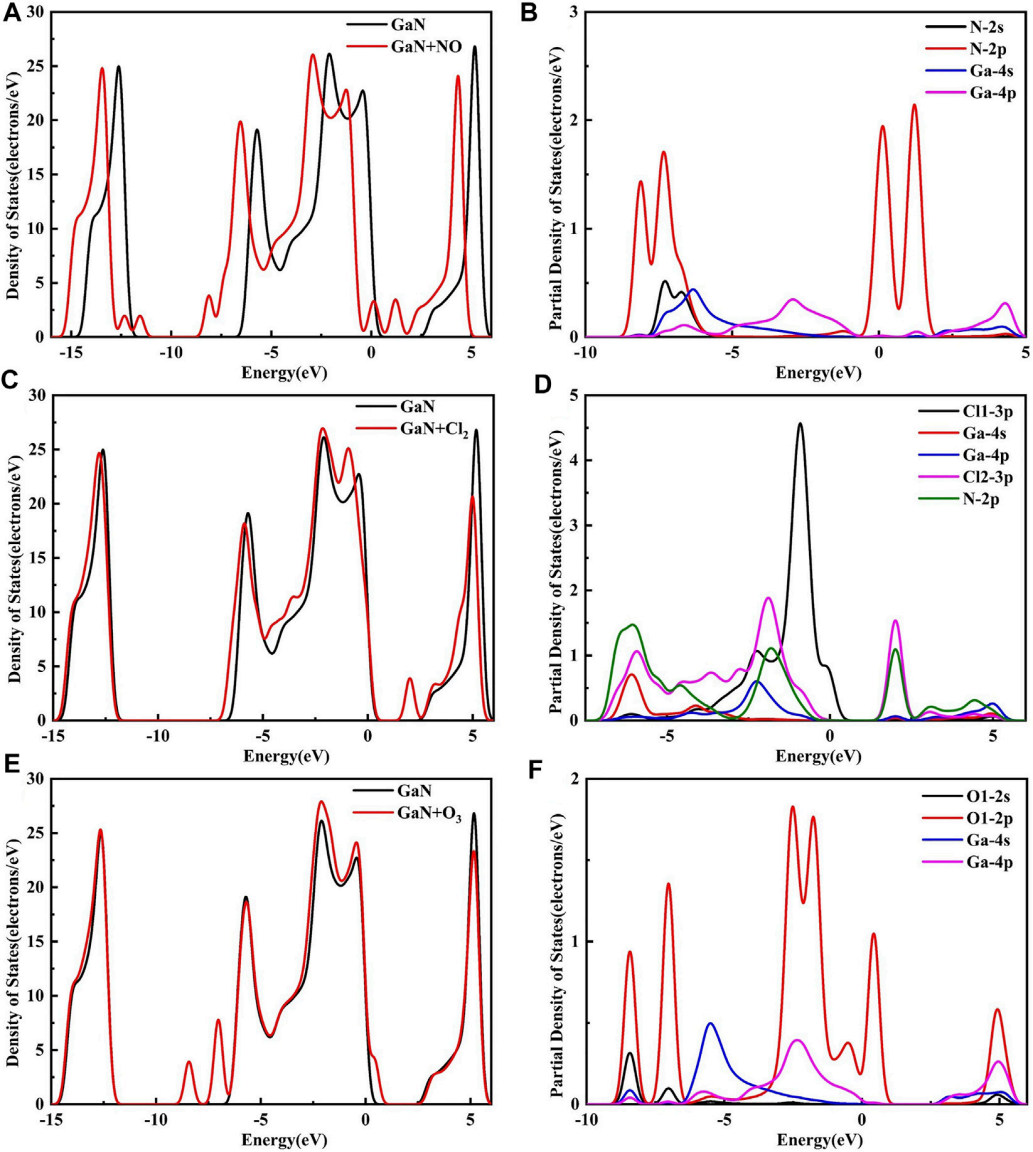
DOS for **(A)** NO, **(C)** Cl_2_, and **(E)** O_3_ on the GaN surface and PDOS for **(B)** NO, **(D)** Cl_2_, and **(F)** O_3_ on the GaN surface.

**FIGURE 9:**
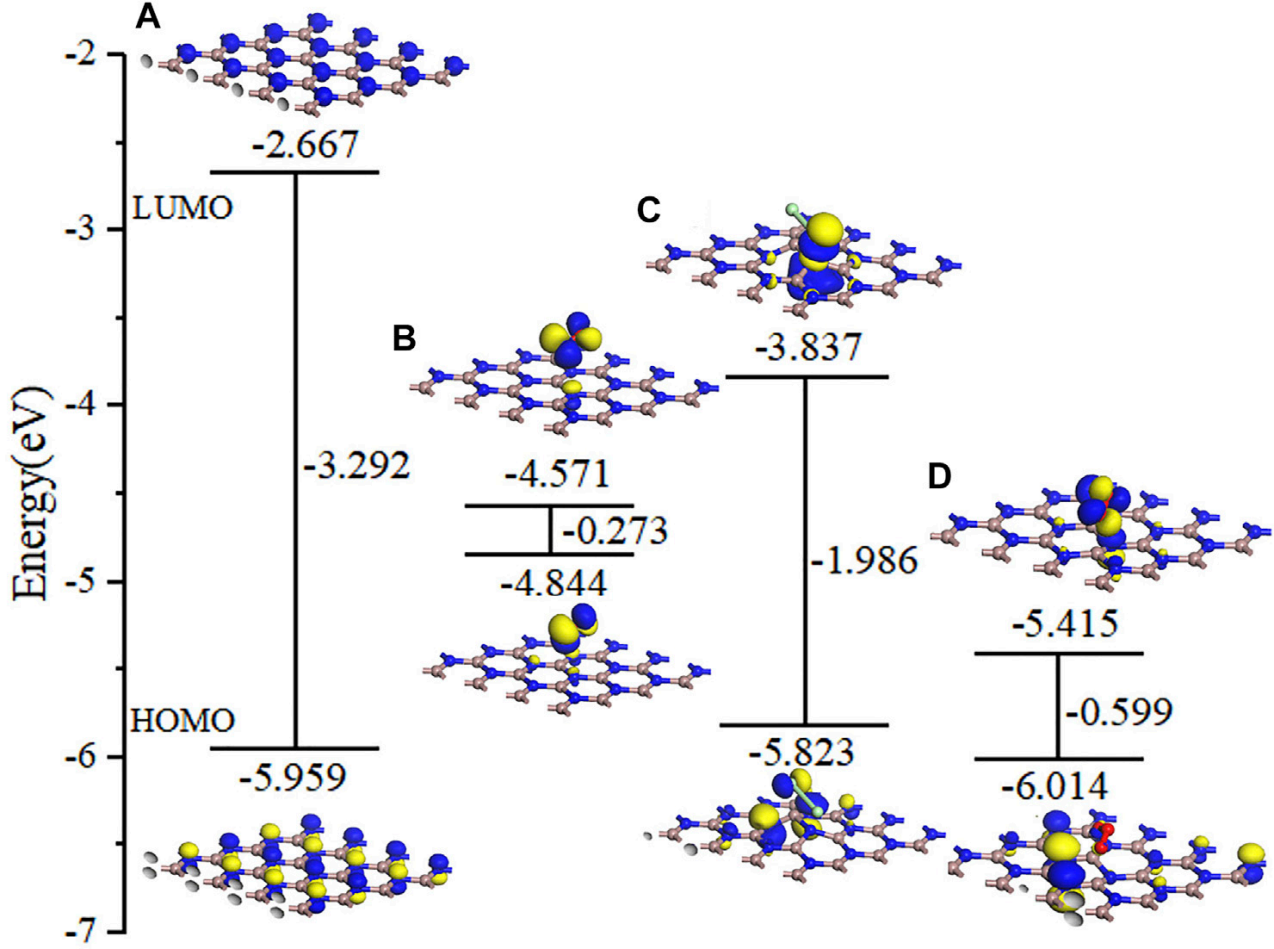
Molecular orbital for **(A)** GaN monolayer, **(B)** NO, **(C)** Cl_2_, and **(D)** O_3_ on the GaN surface.

Figure 8 shows the DOS and PDOS of the three molecules on intrinsic GaN. Compared with intrinsic GaN, magnetism is generated in the whole NO adsorption system in Figure 8A. After NO molecule is adsorbed, two energy peaks appear near the Fermi level of the DOS, which are due to the asymmetric spin-up and spin-down energy levels induced by the N-2p orbital. Therefore, the introduction of NO gas molecule makes the whole adsorption system fail to achieve a stable structure, so magnetism is generated. The unstable adsorption structure can also be demonstrated by the PDOS in Figure 8B with almost no overlapping peaks of each atomic orbital. The DOS of the Cl_2_ adsorption system shows an energy peak elicited by Cl_2_-3p on the right side of the Fermi level, thus causing changes in the band gap and the crystal surface conductivity. According to the band gap calculated by HOMO and LUMO in the molecular orbital diagram in Figure 9, it can also be proved that the GaN crystal plane exhibits a significantly increased response when it absorbs O_3_ molecule. Combining the geometric structure of GaN adsorption on Cl_2_ molecule with PDOS in Figure 8D, it can be observed that a Ga atom of GaN has adsorbed the Cl_1_ atom, so the Cl_1_-3p orbital interacts with the Ga-4s orbital at -6.09 eV, and the Cl_1_-3p orbital has overlapping peaks with Ga-4p at -2.26 eV. An N atom of GaN adsorbed Cl_2_, resulting in overlapping peaks of Cl2-3p and N-2p occurring at positions of -6.02 eV, -1.90, 2.01, and 3.12 eV, which indicates that stable Cl_1_–Ga and Cl_2_–N bonds are formed. Figure 8F shows a significant overlap between Ga and O_1_ orbits in -8.41 eV, -2.51eV, and 4.93 eV, symbolizing that chemical reactions occurred. By simultaneously comparing the band gap in Figures 9A,C, *E*_g_ of GaN sharply decreased after absorbing O_3_. In a word, the analysis of the gas adsorption properties and the conductivity shows that GaN is sensitive to Cl_2_ and O_3_ gases.

**FIGURE 10:**
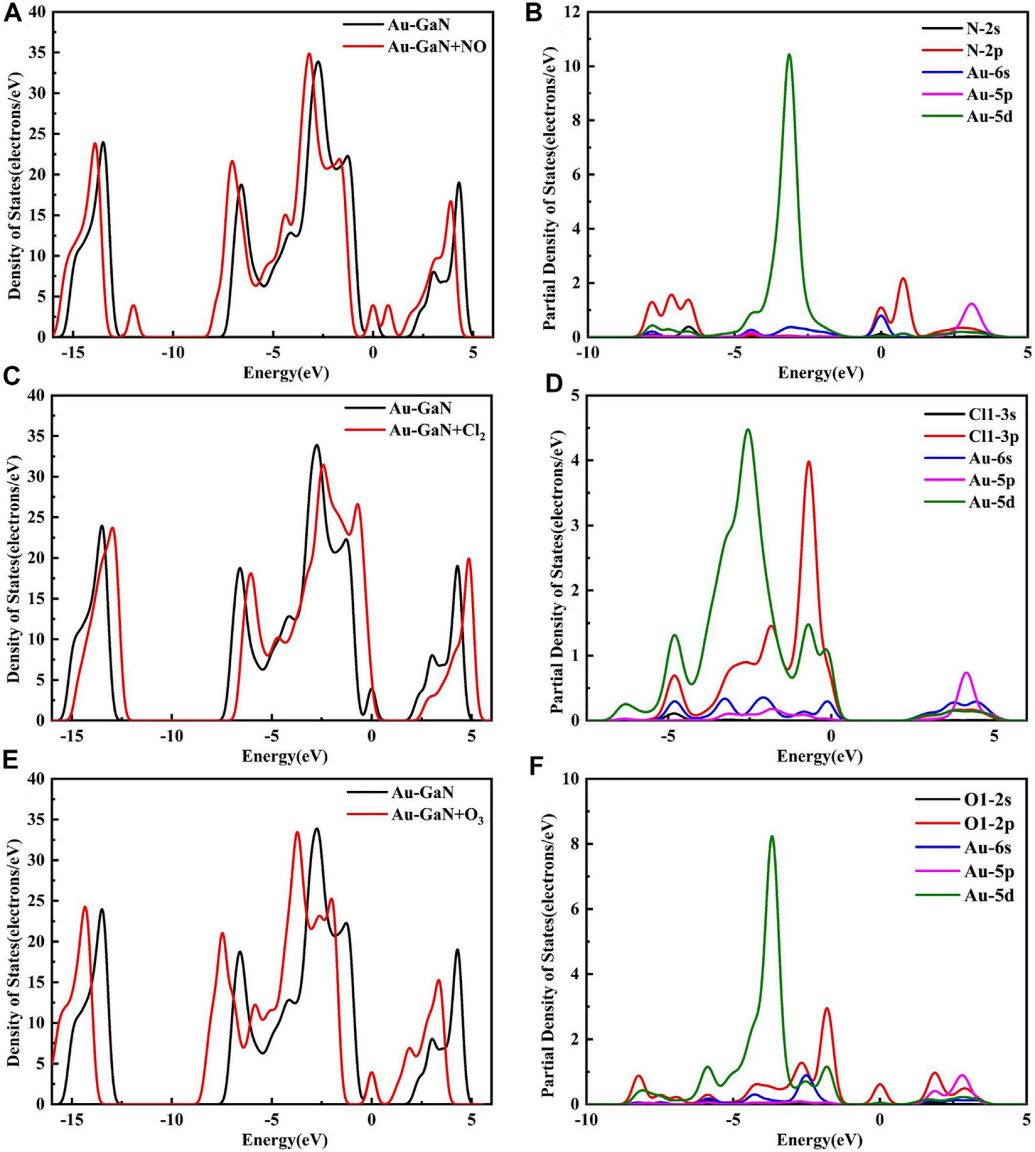
DOS for **(A)** NO, **(C)** Cl_2_, and **(E)** O_3_ on the Au–GaN surface and PDOS for **(B)** NO, **(D)** Cl_2_, and **(F)** O_3_ on the Au–GaN surface.

**FIGURE 11:**
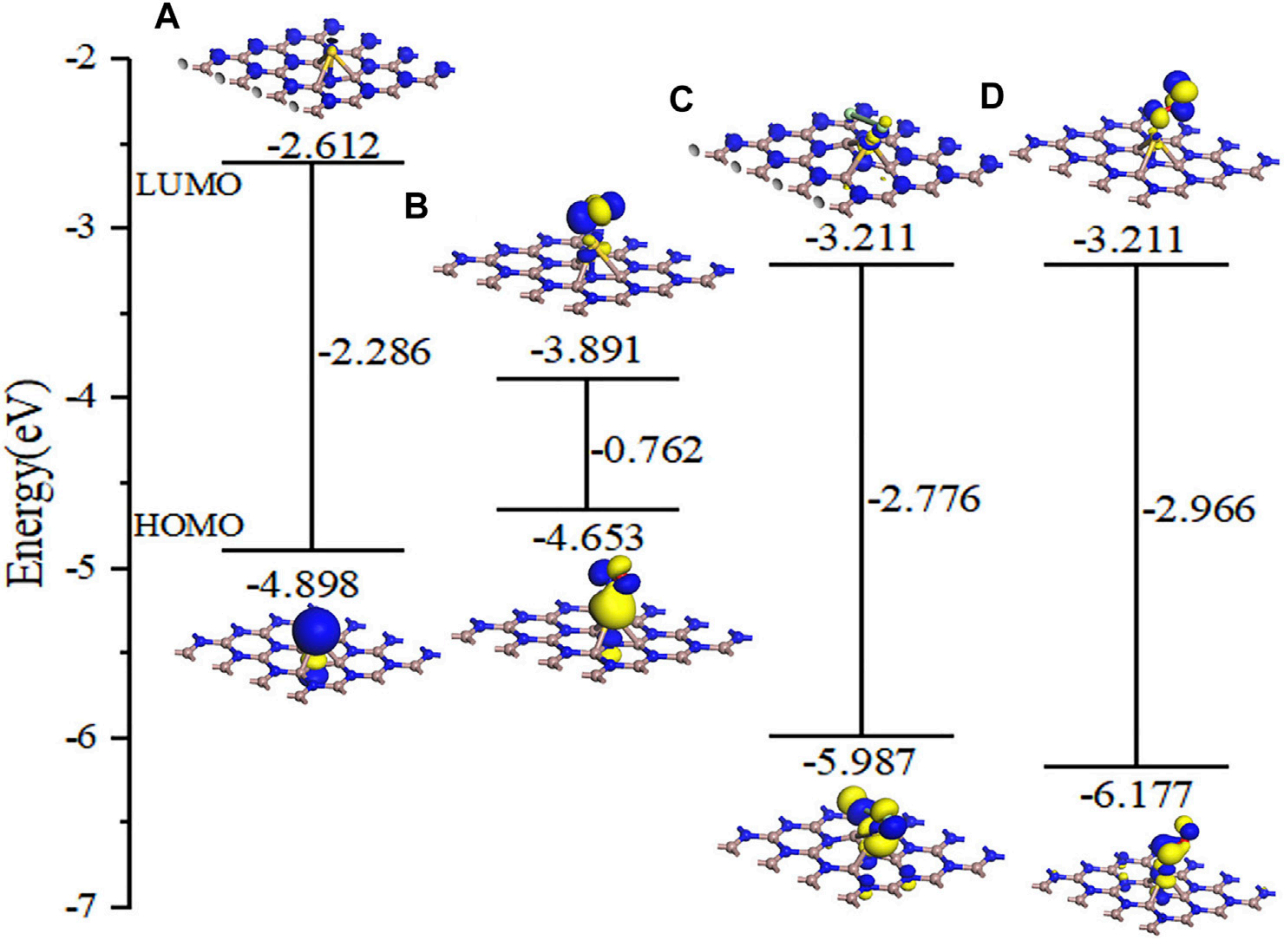
Molecular orbital for **(A)** Au–GaN monolayer, **(B)** NO, **(C)** Cl_2_, and **(D)** O_3_ on the Au–GaN surface.

It can be seen from Figure 10A that although a new energy domain is generated at the Fermi level for NO adsorption on Au–GaN, the symmetry of the whole adsorption system is not damaged, and magnetism is not generated. Moreover, compared with the PDOS of NO adsorbed by the intrinsic GaN, NO has a strong interorbital hybridization with Au–GaN, which is reflected in the obvious overlap between N-2p and Au-5d at -7.81 eV and -3.14 eV and between N-2p and Au-6s at -0.01eV in Figure 10B. In Figure 10D, PDOS shows strong orbital hybridization in the Cl_2_ adsorption system in the range of -5.65 eV ∼ -0.45 eV. In Figure 10E, the DOS of the O_3_ system shows an obvious peak in the Fermi level region, suggesting an increase in the number of conduction electrons provided to the adsorption system. Meanwhile, we observe more PDOS overlapping peaks in Figure 10F, which also demonstrates the stability of adsorption. In Figure 11, based on the rule of band gap change in the adsorption process, the change rule of electrical conductivity can be judged, and then the three gases can be selectively detected using sensitive materials. According to the theoretical calculation, the results show that the conductivity of Au-decorated GaN decreases in different degrees after adsorption of Cl_2_ and O_3_ but increases obviously after the adsorption of NO, which further proves that the sensor prepared by Au–GaN has good sensitivity to these three gases.

## Conclusions

The research in this study is mainly conducted by using simulation technology, and the stability models of three harmful gases (NO, Cl_2_, and O_3_) and Au-decorated GaN were established by DFT. The gas sensitivity mechanism of the three gases near the intrinsic GaN and Au-decorated GaN monolayer was studied, and the following main conclusions were drawn: the most stable configuration of the Au-decorated GaN is that the Au atoms tend to be adsorbed at a perpendicular position directly above the N atoms on the GaN surface. In contrast to the physical adsorption of NO by the intrinsic GaN, the adsorption of NO by the Au-decorated GaN is converted to chemisorption. Therefore, combined with the sensitivity of adsorption and the change in electrical conductivity before and after adsorption, it can be speculated that intrinsic GaN can serve as a gas sensor material for Cl_2_, O_3_, and Au–GaN as a gas-sensitive material for the harmful gas NO. Compared with intrinsic GaN, Au–GaN interacts more strongly with Cl_2_ and O_3_, as reflected by more than three-fold increase of Cl_2_ and more than two times increase of O_3_ in the adsorption energy. Therefore, it can be speculated that Au-decorated GaN can serve as an adsorbed cleaning material for toxic gases Cl_2_ and O_3_. To a large extent, the simulation study in this article carries out the prediction and guidance experiment, which provides a certain prospect for the future application of GaN and Au-doped GaN in gas sensors and toxic gas sorbents.

## Data Availability

The original contributions presented in the study are included in the article/Supplementary Material, further inquiries can be directed to the corresponding author.
